# An analysis of the learning styles and attitudes of foreign students in a post-baccalaureate medical education program

**DOI:** 10.1186/s12909-023-04487-8

**Published:** 2023-07-05

**Authors:** Hsiang-Chin Hsu, Tzu-Ching Sung

**Affiliations:** 1 Department of Emergency Medicine, National Cheng Kung University Hospital, College of Medicine, National Cheng Kung University, Tainan, Taiwan; 2 School of Medicine, College of Medicine, National Cheng Kung University, Tainan, Taiwan; 3 Department of Emergency Medicine, College of Medicine, National Cheng Kung University, Tainan, Taiwan; 4 School of Medicine for International Students, College of Medicine, I-Shou University , No. 8, Yida Rd., Jiaosu Village, Yanchao District, Kaohsiung City, 82445 Taiwan

**Keywords:** Learning styles and attitudes, Post-baccalaureate, Medical education

## Abstract

In a scenario of ongoing changes in the theory and methodology of teaching, student-centered practices are crucial in improving teaching and learning outcomes. This study aimed to evaluate whether the learning styles and attitudes (connected and separate knowing) associated with the curriculum differ among medical students. The research subjects consisted of 43 first- and second-year medical students attending a post-baccalaureate medical education program exclusively for foreign students at a comprehensive university in Kaohsiung City, Taiwan. A self-administered Attitudes Toward Thinking and Learning Survey (ATTLS) was used to assess the differences in learning styles and attitudes among grades, gender, and nationality of these post-baccalaureate medical students. The reliability value of Cronbach Alpha coefficients for all items of ATTLS was 0.93. These medical students reported significantly higher connected knowing styles than separate knowing. The average score of the connected knowing for first-year students taking the "International Health" course is significantly higher than that of second-year students taking the "Population Health and Sustainable Development" course. There is no difference in the separate knowing between these two curricula. The learning styles and attitudes of students participating in the teaching process showed no difference in grade, gender, and nationality. The evidence that there is a significant interaction effect of grade, gender, and nationality examined with the separate knowing, rather than the connected knowing, suggests that this heterogenicity of learning methodology needs to be considered and integrated into future teaching methods.

## Introduction

Medical teaching is an evolving and modernizing process that substantially requires that both students and teachers continuously reflect and upgrade themselves to improve learning and teaching [1]. This is a huge challenge, as teachers must impart a large amount of medical knowledge within a limited time frame so that students can not only remember and understand but also be able to interpret and apply their knowledge for future internships or clinical practice. This has led to a crucial shift for most medical schools needing to reorganize their curriculum and adopt new methods of learning and teaching to varying degrees from traditional didactic teacher-oriented and basic subject learning-centered teaching to interactive, student-centered, or problem-based teaching methods [1, 2]. Researchers suggested that this approach to learning styles was beneficial for both teachers and students because teachers could adjust pedagogy according to student’s learning styles and students would be empowered to use the best techniques suited to their learning styles, resulting in better educational satisfaction and better learning outcomes [2, 3].

Learning is the process whereby knowledge is created through the transformation of experience [4]. Each student has his unique way of learning to acquire, process, memorize and recall information. Learning styles differ from one learner to another and each has specific attributes to learning and the methods by which they can cope with the learning environment. Students may adapt one or more preferred learning styles to acquire new knowledge. Learning styles also play a major role in students' preference for certain teaching approaches and learning environments [5]. Learners consistently have styles that they believe are reasonable enough to apply in their setting to help them attain the most positive learning outcomes [6]. Although there are many different types of learning styles with different conceptual models, the Attitudes Toward Thinking and Learning Survey (ATTLS) model is one of the most common ones, designed to help learners better understand their personal learning preferences [7]. Procedural knowing, which is the process of obtaining, reacting to, evaluating, and communicating knowledge, has been given the greatest attention in ATTLS. Belenky et al. identified two distinct types of procedural knowledge, which they called "separate" and "connected" knowing [8]. In a separate knowing, something is objectively, analytically, and detachedly evaluated, often thought of as being the same as "thinking." In contrast, connected knowing involves aligning oneself with another's point of view, even if it differs from one's own. The concept of procedural knowledge (also known as practical knowledge, imperative knowledge, or performative knowledge) refers to knowledge that is used to perform a task.

Medical education teachers are entrusted with a mission to emphasize individual differences and to effectively achieve their learning needs. Understanding learning styles and attitudes will help teachers develop appropriate approaches to their students' education-orientated approach to the preferred method of learning. Students learning processes are determined by their differences in learning, which is an important factor in improving their academic performance [2, 9]. Learning styles and attitudes vary according to students' tendency to understand, consolidate, process information, and acknowledge learning experiences. Therefore, each student has different learning strategies and attitudes based on their distinct individual differences. Different learning methods have been proposed to determine students' learning styles. Learning in humans is a complex phenomenon that encompasses a wide range of cognitive processes. In recent decades, various theoretical models have been developed to explain it. Among these models, the Experiential Learning Theory (ELT) proposed by David Kolb in 1984 has been particularly influential. This theory posits that knowledge acquisition through direct experience or active engagement is a more effective approach to learning [10]. Both Kolb's (1984) learning stages and cycles could be used by teachers to critically evaluate the learning provision typically available to students and to develop more appropriate learning opportunities and strategies to improve their learning outcomes [4]. Medical teachers should ensure that activities are designed and carried out in ways that offer each student the chance to engage in the manner that suits them best. Medical students can also be helped to learn more effectively by the identification of their lesser preferred learning styles and the strengthening these through the application of the learning cycle.

Launched in 2013, the bachelor's degree in medicine scholarship for foreign students is funded by the International Cooperation and Development Fund (ICDF), the foremost foreign aid organization in Taiwan. Participants attend the School of Medicine for International Students (SMIS) of I-Shou University, a division of the university’s College of Medicine established specifically for the initiative. The SMIS curriculum is divided into two stages. For their first two years, students take general education and medical science curricula at ISU in fields ranging from genetics and human morphology to microbiology and physician–patient communications. All the lessons are conducted in English, except for mandatory Mandarin language classes. In the final two years, students complete clinical clerkships by participating in rounds and outpatient care at one of two nearby healthcare facilities, E-Da Hospital, and E-Da Cancer Hospital. Both institutions, along with ISU, are affiliated with the Kaohsiung-based conglomerate E United Group [11].

This is a unique post-baccalaureate medical education program exclusively for foreign students in Taiwan. In the context of ongoing changes in the theory and methodology of teaching, student-centered practices are crucial in improving teaching and learning outcomes and are especially important for students from many countries. Thus, the objectives of this study are to describe and explore learning styles and attitudes toward curricula among post-baccalaureate medical students during the first two years of their participation in the teaching process and to investigate how different factors such as students' grades, gender, and nationality on the connected and separate knowing of learning styles and attitudes toward the medical science curricula. By understanding these factors, the study aims to contribute to the improvement of teaching and learning outcomes for foreign medical students in Taiwan.

## Methods

**Fig. 1:**
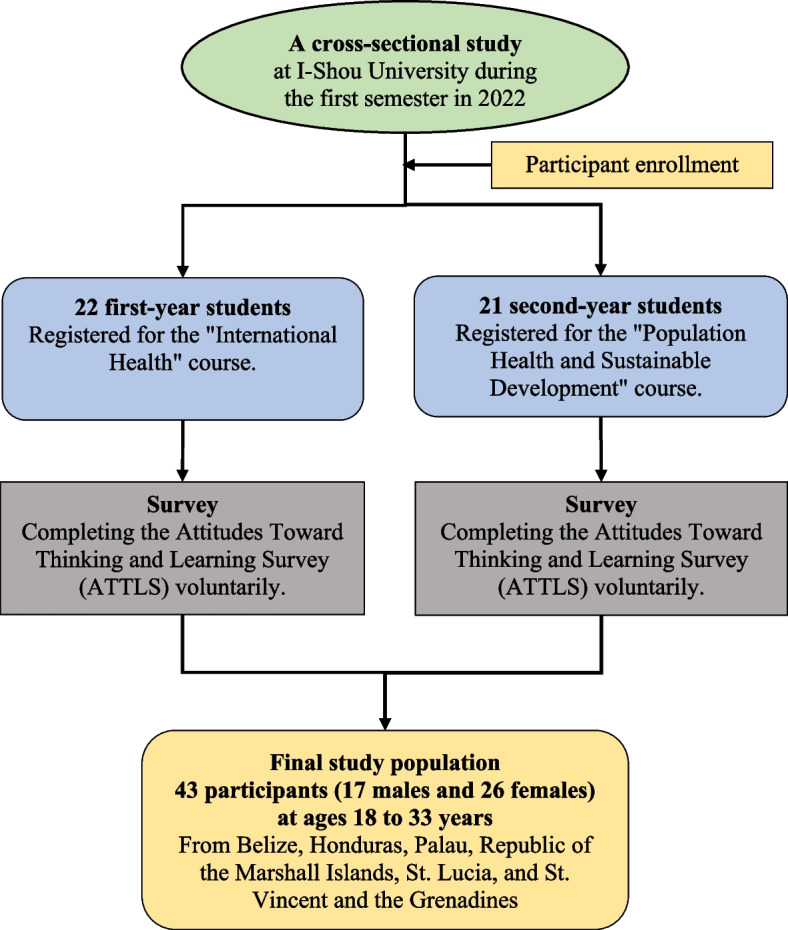
Flowchart of study design and data collection procedure

This study was a descriptive, cross-sectional study that was designed to investigate the self-perception of learning styles and attitudes of post-baccalaureate medical students in a post-baccalaureate medical education program at I-Shou University in Kaohsiung, Taiwan. The program is unique in that it caters to foreign students from allied nations in Africa, the Pacific islands, Central and South America, and the Caribbean, intending to equip them with the necessary knowledge and skills to become competent and compassionate healthcare professionals. The curriculum of the program covers various medical fields, including international health, population health, and sustainable development. The design of the study and the procedure for collecting data are presented in Fig. 1.

### Setting and participants

The study population consisted of all first- and second-year students attending a post-baccalaureate medical education program exclusively for foreign students at I-Shou University who had been taught in curricula on "International Health" and "Population Health and Sustainable Development" during the first semester of 2022.

During the same term, we included in our study all first-year post-baccalaureate medical students who were registered in the "International Health" course and second-year students who were enrolled in the "Population Health and Sustainable Development", and we ensured that their language proficiency and academic performance were comparable to those assessed in the admission examination. The conditions and curriculum for both courses were very similar and repetitive, maintained by the same professor. Both courses are mandatory, and each course carries a credit value of two. The purposive sampling approach was utilized to enroll a total of 43 participants (consisting of 17 males and 26 females) who originate from different nations and diverse backgrounds, such as Belize, Honduras, Palau, the Marshall Islands, St. Lucia, and St. Vincent and the Grenadines. The participants are between the ages of 19 to 33 years old and have completed their undergraduate studies in a range of fields, including biology, chemistry, and public health.

### Measurement tool

The Attitudes Toward Thinking and Learning Survey (ATTLS) is a well-established tool that measures college students' attitudes toward learning and thinking [7]. It is a 50-item survey that evaluates "separate knowing" and "connected knowing" aspects of learning. Separate knowing refers to the idea that knowledge is acquired through objective analysis and logical reasoning, while connected knowing suggests that knowledge is acquired through personal experience and intuition. The ATTLS was designed to assess the ways individuals acquire and process information that has been utilized in studies to evaluate learning styles and gender differences in online learning [12]. Moreover, it has been applied to appraise the effectiveness of educational programs in developing critical thinking and problem-solving skills. Several studies have established that the ATTLS is a reliable and valid tool to measure students' attitudes and learning styles related to critical thinking and problem-solving [7, 12].

To minimize disruption to the class's progress, conserve time and resources, and swiftly yield insights into students' learning attitudes and thinking in the classroom, we utilized a condensed version of the Attitudes Toward Thinking and Learning Survey (ATTLS) survey to evaluate learning attitudes and styles towards critical thinking and problem-solving in the courses of "International Health" and "Population Health and Sustainable Development" as students' participation in the teaching process and that it is highly correlated with the original 50-item version and is nearly as reliable [7].

Students were asked to choose one of five statements on a five-point Likert scale (strongly disagree, disagree, neutral, agree, and strongly agree). The instrument consists of 20 statements, 10 expressing statements exemplifying connected knowing (e.g., “When I encounter people whose opinions seem alien to me, I make a deliberate effort to 'extend' myself into that person, to try to see how they could have those opinions,” and “I'm more likely to try to understand someone else's opinion than to try to evaluate it”) and 10 items exemplifying separate knowing (e.g., “I like playing devil's advocate—arguing the opposite of what someone is saying,” and “In evaluating what someone says, I focus on the quality of their argument, not on the person who's presenting it”). The two types of statements were intermixed in the survey.

### Data collection procedures

The school year consists of two semesters, with 18 weeks each semester. Students were encouraged to fill in the questionnaire in the eighth week in November after the 2022 semester begins. The participation was entirely voluntary and by completing the survey, one was considered to have given informed consent. Students confirmed that the purpose of this questionnaire was to help them evaluate their attitudes toward and learning in class discussions. There are no 'right' or 'wrong' answers, only valuable opinions. Students must be assured that their responses will be treated with a high degree of confidentiality and will not affect their assessments.

In the classroom setting, all 43 students voluntarily completed the ATTLS questionnaire. To safeguard the privacy of the participants, the professor later presented depersonalized data derived from the Moodle system directly, which included descriptive statistics such as frequencies, percentages, means, and standard deviations. This information was presented to the students to provide them with immediate feedback regarding their learning styles and attitudes.

### Statistical analysis

Descriptive statistics were mainly evaluated for demographics and overall responses, and they included frequencies, percentages, means, and standard deviation (SD). Frequencies and percentages were used to describe the distribution of categorical variables such as gender, nationality, and grade. Means and standard deviations were used to summarize the distribution of continuous variables such as age, and the scores for the different learning styles and attitudes. In addition, the Cronbach Alpha reliability coefficient test was used to determine the reliability and assess the consistency of the items in the ATTLS that measured the different learning styles and attitudes.

Furthermore, a non-parametric statistical test, the Mann–Whitney U Test (also known as the Wilcoxon Rank Sum Test), was used to compare two aspects of connected knowing and separate knowing median scores. This test was used because our data did not meet the assumption of normality required for parametric tests such as t-tests. Moreover, multivariate ANOVA (MANOVA) which extends the capabilities of analysis of variance (ANOVA) by assessing multiple dependent variables simultaneously was applied to explore the effect of grade, gender, and nationality on students' learning styles and attitudes toward participation in the teaching process. MANOVA allowed us to test whether the scores for the different learning styles and attitudes vary significantly between groups while controlling for other variables. We used MANOVA to overcome the limitations of univariate ANOVA, which can only assess one dependent variable at a time. Finally, all statistical analyses were performed using the Statistical Packages for Social Sciences (SPSS) (version 27.0) (IBM Corp., Armonk, N.Y., USA). The *p*-value for statistical significance was set at 0.05.

### Ethics statement

The study was approved by I-Shou University and the Institutional Review Boards (IRB) of the collaborating hospitals (IRB No. 2022020). 

## Results

Table 1 provides demographic information about the participants. The study involved 43 students, 60.5% (26 students) of whom were female, and 39.5% (17 students) were male. Regarding the students' grade level, 51.2% (22 students) were first-year students, and 48.8% (21 students) were second-year students. The average ages for first-year and second-year students are 25.1 (ranging from 19 to 33) and 27.1 (ranging from 22 to 33). As for nationality, most of the students were from St. Lucia (39.5%), followed by Belize (34.9%), including countries as follows: Belize (15 students); Honduras (7 students); Palau (1 student); Republic of the Marshall Islands (2 students); St. Lucia (17 students); and St. Vincent and the Grenadines (1 student).

**Table 1:** Demographic data of the participants

	Number	Percent (%)
Gender		
Female	26	60.5
Male	17	39.5
Grade		
First-year	22	51.2
Second year	21	48.8
Nationality		
Belize	15	34.9
Honduras	7	16.3
Palau	1	2.3
Republic of the Marshall Islands	2	4.7
St. Lucia	17	39.5
St. Vincent and the Grenadines	1	2.3

Table 2 shows the reliability values of the learning styles and attitudes measured in the study. The connected and separate knowing scales, each with ten items, had reliability values of Cronbach Alpha coefficients of 0.93 and 0.85, respectively. The total scale, which includes both connected and separate knowing items, had a Cronbach's Alpha value of 0.93, indicating high internal consistency.

**Table 2:** Reliability value regarding the learning styles and attitudes

Aspect	No. of Items	Cronbach's alpha
Connected	10	0.93
Separate	10	0.85
Total	20	0.93

Table 3 presents the descriptive statistics of the learning styles and attitudes measured in the study, stratified by grade, gender, and nationality. Whether first-year students taking the “International Health” course or second-year students taking the “Population Health and Sustainable Development” course, the connected knowing scores are higher than the separate knowing scores. The average of connected knowing (4.2) among first-year students in the “International Health” course is significantly higher than second-year students (4.0) in the “Population Health and Sustainable Development” course. For connected knowing, the mean scores ranged from 3.7 to 4.8 out of 5, indicating a positive attitude towards the medical science curricula. The highest mean score was obtained by first-year female students from Belize, and the lowest mean score was obtained by second-year male students from Honduras. The two-sided *p*-value was 0.0336, indicating a statistically significant difference between the mean scores of the first-year female students from Belize and the second-year female students from Belize.

**Table 3:** Descriptive statistics of the learning styles and attitudes by grade, gender, and nationality

Aspect	Grade	Gender	Nationality	Mean	SD	N	Grade	Gender	Nationality	Mean	SD	N	Two-sided *p* value^*^
Connected knowing	First-year	Female	Belize	4.8	0.2	5	Second year	Female	Belize	4.2	0.3	4	0.0336
			Honduras	4.6	0.0	2			Honduras	3.7		1	
			St. Lucia	3.8	1.2	8			St. Lucia	4.1	0.4	5	
									St. Vincent and the Grenadines	4.1		1	
			Summary	4.2	1.0	15			Summary	4.1	0.3	11	
		Male	Belize	4.7	0.4	3		Male	Belize	3.9	0.8	3	
			Honduras	2.9		1			Honduras	4.0	0.1	3	
									Palau	4.3		1	
			Republic of the Marshall Islands	4.1		1			Republic of the Marshall Islands	4.1		1	
			St. Lucia	4.3		1			St. Lucia	3.3	2.0	3	
			Summary	4.2	0.7	6			Summary	3.8	1.0	11	
		Total	Belize	4.7	0.3	8		Total	Belize	4.1	0.5	7	
			Honduras	4.0	1.0	3			Honduras	4.0	0.2	4	
									Palau	4.3		1	
			Republic of the Marshall Islands	4.1		1			Republic of the Marshall Islands	4.1		1	
			St. Lucia	3.8	1.1	9			St. Lucia	3.8	1.2	8	
									St. Vincent and the Grenadines	4.1		1	
			Summary	4.2	0.9	21			Summary	4.0	0.8	22	
Separate knowing	1	Female	Belize	3.7	0.4	5	2	Female	Belize	3.9	0.6	4	0.5540
			Honduras	3.8	0.4	2			Honduras	2.6		1	
			St. Lucia	3.2	0.8	8			St. Lucia	3.1	0.3	5	
									St. Vincent and the Grenadines	4.2		1	
			Summary	3.4	0.7	15			Summary	3.5	0.7	11	
		Male	Belize	3.9	0.3	3		Male	Belize	2.9	1.3	3	
			Honduras	2.7		1			Honduras	3.7	0.4	3	
									Palau	3.5		1	
			Republic of the Marshall Islands	3.3		1			Republic of the Marshall Islands	3.4		1	
			St. Lucia	4.7		1			St. Lucia	3.0	1.8	3	
			Summary	3.5	0.7	21			Summary	3.2	1.1	11	
		Total	Belize	3.8	0.4	8		Total	Belize	3.5	1.0	7	
			Honduras	3.4	0.7	3			Honduras	3.4	0.6	4	
									Palau	3.5		1	
			Republic of the Marshall Islands	3.3		1			Republic of the Marshall Islands	3.4		1	
			St. Lucia	3.4	0.9	9			St. Lucia	3.1	1.0	8	
									St. Vincent and the Grenadines	4.2		1	
			Summary	3.5	0.7	21			Summary	3.4	0.9	22	

^*^Mann Whitney U Test (Wilcoxon Rank Sum Test)

However, there is no difference in the separate knowing between these two curricula for the students. For separate knowing, the mean scores ranged from 2.6 to 4.7 out of 5, indicating a diverse attitude towards the medical science curricula. The highest mean score was obtained by second-year male students from St. Lucia, and the lowest mean score was obtained by first-year male students from Belize. The two-sided p-value was 0.5540, indicating no significant difference between the mean scores of the groups.

The MANOVA results in Table 4 show the effects of grade (Wilk's Ʌ = 0.452, F = 0.485, *p* = 0.910, effect size Eta-squared (η2) = 0.548), gender (Wilk's Ʌ = 0.149, F = 2.284, *p* = 0.116, η2 = 0.851), or nationality (Wilk's Ʌ = 0.004, F = 0.944, *p* = 0.602, η2 = 0.675) have consistent across the different items of students' learning styles and attitudes toward participation in the teaching process. There was a statistically significant 3-way interaction effect of grade, gender, and nationality on one connected and three separate knowing items of ATTLS. The interaction effect of gender and nationality on the connected knowing is not the same for the first- and second-year students taught in curricula on “International Health” and “Population Health and Sustainable Development”, i.e., “I am always interested in knowing why people say and believe the things they do.” (Type III Sum of Squares (SS) = 8.354, F = 3.422, *p* = 0.047, η2 = 0.202). The interaction effects of gender and nationality on three separate knowing items are not the same for the first- and second-year students attending these two curricula, either: “I find that I can strengthen own position through arguing with someone who disagrees with me.” (Type III SS = 32.685, F = 10.173, *p* = 0.001, η2 = 0.430), “I often find myself arguing with the authors of books that I read, trying to logically figure out why they're wrong.” (Type III SS = 7.610, F = 3.553, *p* = 0.043, η2 = 0.208), and “I try to point out weaknesses in other people's thinking to help them clarify their arguments.” (Type III SS = 15.283, F = 3.951, *p* = 0.031, η2 = 0.226).

**Table 4:** Multivariate analysis of learning styles and attitudes by grade, gender, and nationality

The aspect of learning styles and attitudes	*P* value of Wilks' Lambda statistic Λ						
	Grade	Gender	Nationality	Grade* Gender	Grade* Nationality	Gender* Nationality	Grade* Gender* Nationality
Connected knowing items	0.504	0.359	0.682	0.821	0.818	0.809	0.216
1. When I encounter people whose opinions seem alien to me, I make a deliberate effort to 'extend' myself into that person, to try to see how they could have those opinions	0.903	0.186	0.411	0.332	0.156	0.365	0.281
2. I can obtain insight into opinions that differ from mine through empathy	0.938	0.171	0.459	0.717	0.718	0.644	0.569
3. I tend to put myself in other people's shoes when discussing controversial issues, to see why they think the way they do	0.357	0.845	0.460	0.850	0.907	0.857	0.723
4. I'm more likely to try to understand someone else's opinion than to try to evaluate it	0.411	0.080	0.218	0.718	0.634	0.901	0.864
5. I try to think with people instead of against them	0.418	0.380	0.665	0.644	0.737	0.671	0.478
6. I feel that the best way for me to achieve my own identity is to interact with a variety of other people	0.779	0.967	0.431	0.116	0.972	0.563	0.164
7. I am always interested in knowing why people say and believe the things they do	0.546	0.152	0.772	0.847	0.445	0.613	0.047
8. I enjoy hearing the opinions of people who come from backgrounds different to mine—it helps me to understand how the same things can be seen in such different ways	0.553	0.600	0.924	0.657	0.641	0.717	0.670
9. The most important part of my education has been learning to understand people who are very different to me	0.411	0.965	0.607	0.883	0.776	0.949	0.074
10. I like to understand where other people are 'coming from', what experiences have led them to feel the way they do	0.481	0.957	0.877	0.415	0.948	0.850	0.448
Separate knowing items	0.326	0.776	0.816	0.699	0.762	0.248	0.080
11. I like playing devil's advocate—arguing the opposite of what someone is saying	0.352	0.105	0.229	0.659	0.519	0.424	0.137
12. It's important for me to remain as objective as possible when I analyze something	0.146	0.767	0.990	0.936	0.768	0.196	0.788
13. In evaluating what someone says, I focus on the quality of their argument, not on the person who's presenting it	0.426	0.463	0.903	0.354	0.880	0.527	0.757
14. I find that I can strengthen my own position through arguing with someone who disagrees with me	0.381	0.719	0.877	0.971	0.783	0.964	0.001
15. One could call my way of analyzing things 'putting them on trial' because I am careful to consider all the evidence	0.881	0.391	0.731	0.487	0.517	0.503	0.433
16. I often find myself arguing with the authors of books that I read, trying to logically figure out why they're wrong	0.550	0.564	0.193	0.647	0.243	0.538	0.043
17. I have certain criteria I use in evaluating arguments	0.326	0.670	0.819	0.134	0.787	0.863	0.891
18. I try to point out weaknesses in other people's thinking to help them clarify their arguments	0.757	0.771	0.627	0.293	0.805	0.640	0.031
19. I value the use of logic and reason over the incorporation of my own concerns when solving problems	0.701	0.407	0.995	0.223	0.604	0.364	0.896
20. I spend time figuring out what's 'wrong' with things. For example, I'll look for something in a literary interpretation that isn't argued well enough	0.463	0.925	0.452	0.688	0.782	0.090	0.174

*represents the interaction term in a MANOVA analysis to examine how the combination of two or more factors jointly affects the dependent variables of interest

Overall, the MANOVA analyses in this study suggest that grade, gender, and nationality are important factors in determining students' learning styles and attitudes toward participation in teaching. It further revealed that the significant 3-way interaction effect of grade, gender, and nationality on one connected and three separate knowing items of ATTLS suggests that the relationship between these factors is more complex than a simple main effect.

## Discussion

The evaluation of ATTLS in our study indicated that the vast majority of students of both genders in this post-baccalaureate medical education program are identified as connected knowers looking for why it makes sense, how it might be right, and then they look at things from the other's point of view and try first to understand the other's point of view rather than evaluate it and place themselves in alliance with another's positions. The findings differed from previous studies in which males were inclined to be separate knowing types and females were inclined to be connected knowing types [13]. The minority of students are separate knowers which implies the issue of objective and analytical, detached evaluation of an argument or piece of work would be potentially involved in designs and methods for further learning evaluations. The lecture design determines whether separate and connected learning styles are applied in the classroom. Cultivating appropriate knowing in the classroom is a teacher's obligation [14]. Medical teachers must use effective pedagogical practice to establish a class as a unit, so they can meet for a productive conversation to take place. Neither students nor teachers will feel threatened or violated during discussions.

Developed by Dr. Sharon Bailin and Dr. Mark David Bailin in the 1990s, the Attitudes Toward Thinking and Learning Survey (ATTLS) is a psychometric instrument that aims to evaluate students' attitudes toward thinking and learning in academic environments [7]. Composed of 50 items that represent "separate knowing" and "connected knowing," the ATTLS measures students' attitudes towards these two modes of learning, where separate knowing refers to the ability to analyze and evaluate information, and connected knowing refers to the ability to integrate and synthesize information [7]. The ATTLS has been employed in numerous studies to evaluate students' attitudes toward thinking and learning [7, 12]. Additionally, it has been used to assess learning styles and gender differences in online learning [12]. The study employed the ATTLS, which is a crucial instrument for educators and researchers aiming to enhance teaching techniques and foster critical thinking abilities by evaluating students' perspectives on learning and thinking in academic environments. Various studies have employed the ATTLS to evaluate the attitudes of students toward thinking and learning [7, 12]. Its effectiveness, reliability, and validity have been assessed in examining teaching methodologies that aim to enhance critical thinking abilities [7]. The ATTLS can be considered a useful tool for educators and researchers who wish to evaluate students' attitudes toward thinking and learning in educational settings, as demonstrated in the study.

The original ATTLS had a 50-item list, so participants would take a long time to administer it (45 min, on average) [7]. The shortened 20-item ATTLS application used in our study has been proven to be highly correlated with the original version with a longer version and nearly as reliable [7]. There is no generally agreed cut-off for Cronbach’s alpha. Nunnally suggested 0.7 and above is acceptable in the early stages of research [15], and he indicated that the required level of Cronbach’s alpha depends on how critical the decisions that are drawn based on the research results, and more stringent cut-offs should be used for basic research (0.80 or higher) and applied research (0.90 or higher) [15, 16]. George and Mallery suggest a tiered cut-off value of the following Cronbach’s alpha: ≥ 0.9 (excellent), ≥ 0.8 (good), ≥ 0.7 (acceptable), ≥ 0.6 (questionable), ≥ 0.5 (poor), and ≤ 0.5 (unacceptable) [17]. Our study shows Cronbach Alpha coefficients for all items and connected aspects (items 1–10) of the learning styles and attitudes were both 0.93, which shows excellent consistency reliability. Regarding separate aspects (items 11–20), the Cronbach Alpha coefficient of 0.85 indicates good reliability. Ensuring good reliability, the consistency of a measure or research instrument, in our study can help to guarantee the trustworthiness and validity of the results [18]. The high-reliability level of the ATTLS survey used in our study increases the confidence we have in the findings [19], thereby reducing the likelihood of random errors or external factors influencing the outcomes.

Our study has emphasized that procedural knowledge is goal-oriented and mediates problem-solving behavior [20]. Schuwirth et al. have demonstrated that problem-solving questions usually assess the application of knowledge (procedural knowledge) rather than simple factual recall (declarative knowledge) [21]. According to cognitive science, knowledge can be divided into 'declarative knowledge' and 'procedural knowledge.' The former is core knowledge, which means 'knowing what'; the latter is executive knowledge, which means 'knowing how'. Essentially, procedural knowledge is about knowing 'how to do it,' as defined by cognitive psychologists. There is a complexity to it that arises from trying to link it to concepts like 'process', 'problem-solving', 'strategic thinking', and the like, which in turn requires different levels of procedure to be distinguished [22]. The ability to execute action sequences helps solve problems. This type of knowledge is tied to specific problem types and therefore is not widely generalizable [23]. However, its advantages are especially apparent in medical curricula. Additionally, feedback and coaching are necessary to help students understand their performance, identify areas for improvement, and make adjustments [24, 25]. In our study, having an experienced professor involved in procedural knowledge training to enhance the learning process. By drawing on their expertise and sharing their insights, these professors can help students develop the necessary skills and knowledge to perform clinical procedures effectively and safely. Their guidance can also help students understand the nuances and complexities of different procedures and provide context and real-world examples that can enhance the learning experience.

The score in the "International Health" course among first-year students is significantly higher than the score of second-year students in the "Population Health and Sustainable Development" course. The lecturer in these two classes is the same person. The "International Health" course was designed to help students spend more time and have opportunities to express themselves in their respective experiences with their own countries. The post-baccalaureate medical students are an international group that includes students from Caribbean countries, including Belize, Honduras, the Republic of the Marshall Islands, and St. Lucia. Different countries' cultural backgrounds and the accumulation of personal experience make them diverse in their attitudes toward learning. As Miyazoe found, learners in different contexts may demonstrate different learning styles and preferences [12]. The content and characteristics of the course also affect the interaction and cohesion of students in the classroom. The course, "International Health," encourages students to engage with others and share their own experiences. Under these conditions and learning objectives, a connective knowledge style, rather than a separate knowledge style, was preferred to strengthen interaction with others and learn more about international health or global issues.

Furthermore, our study revealed a statistically significant 3-way interaction effect of grade, gender, and nationality on one connected and three separate ATTLS items. There was no such effect if we analyzed grade, gender, nationality, or any of the two combined factors. The interaction effect of gender and nationality on the connected knowing item was not the same for the first- and second-year students taught in curricula on “International Health” and “Population Health and Sustainable Development”. This indicates that the effects of gender and nationality on connected knowing may depend on the curriculum or the academic program. Similarly, the interaction effects of gender and nationality on three separate knowing items were not the same for the first- and second-year students attending these two curricula. This suggests that the effects of gender and nationality on separate knowing may depend on the context of the learning environment. For example, students in different programs may have different opportunities to argue with others or to strengthen their positions through debate.

Therefore, medical educators should be cognizant of the varying learning styles and attitudes of their students and modify their teaching techniques and syllabus to accommodate these dissimilarities [26, 27]. Educators might need to offer a variety of instructional resources and exercises that accommodate diverse learning styles of students, including but not limited to, lectures, group conversations, case studies, and practical applications [28]. By establishing a supportive learning environment that involves regular feedback and support, encouraging active participation, and promoting a sense of camaraderie and inclusivity, educators can instill a positive attitude towards medical science curricula among students, as per our study's valuable findings. It also highlighted the significance of heterogenicity in learning methodologies likely resulting from multicultural experiences. Developing medical students' learning attitudes is strongly based on their country's cultural background and personal experience. Their accumulation of experiences in different cultures has resulted in many unique learning attitudes in which experiences can be directly induced into constructive outcomes.

### Limitations

Self-report bias may be a potential limitation of the Attitudes Toward Thinking and Learning Survey (ATTLS) in this study. The social desirability bias may be a concern where participants may overestimate or underreport certain learning styles or attitudes based on what they perceive to be valued by their peers or teachers, or due to fear of negative consequences. However, we have taken measures to mitigate this bias. The teacher in the classroom explained the importance of providing honest responses and ensured the confidentiality and anonymity of the survey. The questionnaire was intended solely for personal reflection on one's true learning attitudes and was not related to any academic evaluation or personal information. We also ensured that the results would be purely presented as a statistical distribution with no personal data, and there would be no comparison with other students. Furthermore, the participants in this study have graduated from university, indicating their maturity in various aspects of learning, and we expect that they would provide honest responses about their attitudes toward thinking and learning.

The ATTLS has certain limitations that may impact its ability to provide a comprehensive picture of students' learning situations. One of the limitations is its limited scope, which only measures certain aspects of learning styles and attitudes while ignoring other factors that may impact learning outcomes. This may lead to biased results and an incomplete understanding of the relationship between student attitudes toward learning and their academic performance. The ATTLS only measures attitudes towards thinking and learning, which includes components of connected and separate knowing, but it does not capture the full range of factors that may impact learning outcomes, such as cognitive abilities, environmental factors, individual differences in learning preferences or styles, and social and cultural factors that may shape attitudes. However, despite these limitations, the ATTLS still offers insights into certain aspects of foreign students' learning styles and attitudes, which can be valuable in our study. The scale has good content validity, high internal consistency reliability is easy to administer and is broadly applicable across different age groups, educational levels, and cultural contexts. These advantages make it a practical tool for research studies and classroom assessments and can provide valuable insights into students' attitudes toward learning. It should not be the only tool used to assess student attitudes toward learning, as it may not provide a complete or comprehensive picture of their learning situations.

The use of nonparametric methods in this study addresses concerns related to the small sample size and helps to mitigate potential biases and unreliable results. Nonparametric tests do not rely on assumptions about the distribution of data and are therefore more robust and flexible than traditional parametric methods [29]. However, despite the use of nonparametric methods, the small sample size in our study remains a limitation. It may have affected the statistical power of our analyses, making it difficult to draw strong conclusions from subgroup analyses. We calculated the post-hoc power of our study based on the sample size of 43 and found that it had only 21% power to detect a small effect size at a significance level of 0.05 which had a low level of power and meant that the possibility of a type II error is indeed a potential limitation of our study. Small sample sizes can be problematic in research because they may lead to biased or unreliable results, as well as increased variability and decreased precision. In addition, small sample sizes may not be representative of the larger population from which the sample was drawn and may not adequately capture the full range of variability within that population. To enhance the reliability and generalizability of findings obtained from subgroup analyses, it is necessary to increase the sample size in future studies. This can help ensure that the results are more robust and less prone to errors. we still believe that our findings contribute to the current literature and provide preliminary evidence for future research.

Our study only included first- and second-year students in medical school at the I-Shou University and that numbers are limited because of training funding. Because the special medical education program exclusively for foreign students in the post-baccalaureate medical department is unique in Taiwan, there is no other school that can be used as a comparison. In some countries only one application per year was included in the analysis, thus limiting the generalization of the findings. Despite these limitations, the results of this study can indeed be used as a reference for future medical education programs intended to recruit foreign students. Our study not only provides valuable insights into the experiences and perceptions of post-baccalaureate medical students in this unique program and may serve as a basis for further research in this area but also the unique challenges and opportunities faced by this population. Future studies with larger sample sizes and more diverse populations may help to validate and expand upon our findings and provide a more comprehensive understanding of the issues and challenges faced by post-baccalaureate medical students in different contexts.

## Conclusion

It is only one post-baccalaureate medical education program exclusively for foreign students in Taiwan that provides valuable insights for medical educators and researchers interested in enhancing the quality of medical education for foreign students. The foreign students preferred a connected, knowing interaction during their lectures. It highlights the importance of enhancing the learning and teaching methodologies to meet the students' preferences and promote intercultural understanding. Therefore, further improvement in learning and teaching methodologies of integrated systems in I-Shou University needs to be done to contribute towards the sustainable development of knowledgeable and highly skilled healthcare in the future. A significant interaction effect of grade, gender, and nationality needs to be further considered and integrated into teaching methods that can ensure that multicultural experiences can be directly induced into constructive outcomes. To obtain a more comprehensive understanding of the challenges faced by post-baccalaureate medical students in various contexts, it may be necessary to conduct future studies with larger sample sizes and more diverse populations. Such studies would enable us to validate and extend our findings. Advanced prospective research into the medical teaching process between Taiwanese medical students and foreign medical students is recommended.

## Data Availability

The datasets used and analyzed during the current study are available from the corresponding author on reasonable request.
